# Effects of Lubricant and Toughening Agent on the Fluidity and Toughness of Poplar Powder-Reinforced Polylactic Acid 3D Printing Materials

**DOI:** 10.3390/polym10090932

**Published:** 2018-08-21

**Authors:** Qingfa Zhang, Hongzhen Cai, Andong Zhang, Xiaona Lin, Weiming Yi, Jibing Zhang

**Affiliations:** 1 School of Agricultural Engineering and Food Science, Shandong University of Technology, Zibo 255000, China; zhangqingfacll@126.com (Q.Z.); chzh666666@126.com (H.C.); antony2362@foxmail.com (A.Z.); linxiaona1120@163.com (X.L.); 2 Shandong Research Center of Engineering and Technology for Clean Energy, Zibo 255000, China; 3 Anhui Aile door and window system Engineering Co., Ltd., Suzhou 234000, China; zhang19730510@163.com

**Keywords:** polylactic acid (PLA), composites, polyolefin elastomer (POE), three dimensional (3D) printing materials

## Abstract

Three dimensional (3D) printing materials were manufactured with polylactic acid (PLA) and poplar powder using the twin screw extruder and 3D printing consumables extruder. Lubricant (TPW604) and toughening agent polyolefin elastomer (POE) were utilized to improve the fluidity and toughness of the materials. 3D printing materials were tested by infrared spectroscopy, X-ray diffraction, melt flow rate, rheology behavior, impact and scanning electron microscope. The results show that the poplar powder could decrease impact strength of PLA, the same as TPW604. Unlike poplar powder, TPW604 can improve the fluidity of 3D printing materials. And POE can fill the voids formed by poplar powder in PLA, enhance interface compatibility between poplar powder and PLA, and effectively improve the fluidity and impact strength of 3D printing materials.

## 1. Introduction

In recent years, three dimensional (3D) printing technology has been developed rapidly in molding and manufacturing of materials and it received more and more attention all over the world because of its special advantages in the making of spatial stereoscopic models [1]. Compared with the industrial material reduction manufacturing, 3D printing technology separates production- manufacturing from large, complex, traditional industrial processes and has changed the manufacturing concepts, processing methods and management models of traditional industry. At present, 3D printing technology has been widely used in aerospace, biomedical, architectural, foundry and other fields [2]. 3D printing technology, also known as additive manufacturing technology or rapid prototyping technology, which is based on the digital models to build a model by adding layer-upon-layer of material [3]. There are four main technologies of 3D printing which include fused deposition modeling (FDM), stereo lithography (SL), selective laser sintering (SLS), layered solid manufacturing (LSM) and so on [4]. The application and development of 3D printing technology mainly depends on 3D printing materials. Metallic, ceramic, polymers and composite materials can be used as 3D printing materials.

So far, FDM is the most widely used in 3D printing technology. However, the materials to be used in FDM must not only meet the requirements of high mechanical strength, low shrinkage, and suitable for melting temperature, but also meet the environmental requirements of safety, non-toxicity, and non-irritating [5]. As one of the materials for FDM, the application of polylactic acid (PLA) is the most common. As a polymer from renewable resources, PLA not only has a wide source of raw material but also can be completely biodegraded. Besides, PLA also could be used in tissue engineering, medical equipment and other 3D printing productions because of its biocompatibility and low toxicity. Other than that, PLA has excellent fluidity and mechanical properties, which make it less prone to breakage during FDM. But there are obvious defects in the properties of PLA which limited the further popularization and application in additive manufacturing technology such as low glass transition temperature, high brittleness and poor thermal stability. In order to remedy the defects and improve the properties of PLA, many scholars have done a lot of research the modification of PLA. Madhavan et al. mixed PLA with polyethylene glycol (PEG) and prepared samples by injecting, the results showed that the elasticity and transparency of PLA have been improved [6]. Fortunati et al. modified PLA with modified cellulose nanocrystals to extrude PLA film, the crystallinity and Young's modulus of PLA increased, because the surface modifier improved the nucleation effect of the PLA composites [7]. Zhang et al. modified PLA with hydroxyapatite, and the modified composite had higher storage modulus than pure PLA [8]. Ferri et al. mixed PLA with another degradable plastic polycaprolactone (PCL), the toughness and degradability of the modified PLA were all improved [9]. In addition to the above, fiber reinforcement is the most common method to modify PLA because of the rich source and the low price. Chun et al. added coconut shell powder to PLA, the tensile strength and thermal stability of the composites were all improved [10]. Hence, some progresses have also been made in the preparation of 3D printing materials by fiber reinforced PLA. Tao et al. used wood flour to fill PLA to make 3D printing materials, wood flour improved tensile strength and thermal stability of PLA [11]. Not only natural fibers, but also carbon fibers are widely used in 3D printing materials. The carbon fiber could also improve the mechanical strength of PLA in 3D printing materials [12,13].

Although the modification of fiber reinforced PLA is very common, the fiber would also have negative impacts on PLA. Yang et al. [14] mixed wood flour with PLA into a biocomposite and hot compression molded. The results showed that wood flour could increase the crystallinity of PLA, but it had a negative effect on its rheology. Patanwala et al. reported 3D printing of carbon nanotube-polylactic acid (CNT-PLA) composites using an extrusion method based on FDM, the overall toughness and fluidity of the composites became worse [15]. Furthermore, the fiber could also reduce the melt index and increase the viscosity of PLA [16], and is not conducive to processing and printing of the composites. As a kind of wood-plastic composite (WPC), wood/PLA composite has the disadvantages of WPC. The wood fiber could effectively improve the mechanical strength of PLA. Wood flour can not only be uniformly dispersed in PLA matrix, but also be effectively encapsulated by PLA macromolecule. But wood fiber would limit the movement of PLA macromolecular chains, which could result in a significant decrease in the crystallization capacity. The impact toughness of wood/PLA composites is poor and brittle fracture would occur when subjected to external force, which is similar to pure PLA. There are two main reasons for this phenomenon [11]: on the one hand, the interfacial compatibility between wood flour and PLA is poor, thus it is difficult to form a stable chemical bond between them, in the other hand wood fiber could generate stress concentration points inside the PLA, which is more likely to cause crack growth and expansion. Hence, it is necessary to toughen wood/PLA composites for improving the toughness and enhancing the strength of 3D printing materials; the toughening modification is generally carried out by the following methods: toughness enhancement of plastic matrix; addition of toughening agent; optimization of wood flour fiber, and so on [17]. Li et al. adopted Surlyn ionomer to improve the interfacial adhesion and impact toughness of the cellulose nanofibers (CNFS)/PLA composite and the composite showed extremely high impact toughness, which was up to 34.2 kJ/m^2^ [18]. Liu et al. used PLA and PCL to prepare 3D printing material with acetyl tributyl citrate (ATBC) as the flexibilizer, and the impact strength of material could reach 10.5 kJ/m^2^ [19].

The objective of this paper is to use wood flour to reinforce PLA to prepare new 3D printing materials, to adopt lubricant and flexibilizer to change the fluidity and toughness. The effects of wood flour, lubricant and flexibilizer on fluidity and impact strength of 3D printing materials are presented and discussed. The research in this paper would provide theoretical guidance for the scientific determination of application fields and provide a theoretical basis for the development of new 3D printing materials.

## 2. Materials and Method

### 2.1. Materials

Poplar powder purchased from Hainuo Co., Ltd. (Shanghai, China) particles were sieved in order to keep them less than 100 μm. PLA (4032D) used was purchased from Yuefa Plastic Co., Ltd. (Dongguan, China) and used as the matrix material. The lubricant (TPW604) was used to reduce the friction between equipment and materials. Polyolefin elastomer (POE) purchased from Hyman Topbon chemical technology Co., Ltd. (Zibo, China) was used as flexibilizer to improve the toughness of 3D printing materials. The poplar powder, PLA and additives were dried in an oven at 105 °C for 24 h prior to keep the moisture content less than 2%.

### 2.2. 3D Printing Materials Fabrication

**Figure 1:**
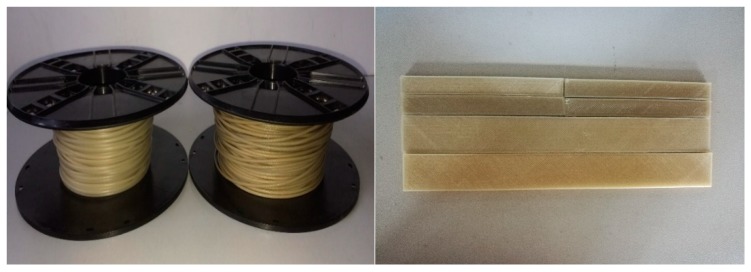
Filamentary three dimensional (3D) printing materials samples and three-dimensional printing plate.

To study the effects of wood flour, lubricant and flexibilizer on fluidity and impact strength of 3D printing materials, 7 concentrations were chosen (Table 1). The raw materials were dry blended by a high speed mixer (JHN-15, Zhengzhou, China) for 10 min to get a homogeneous blend. The dry mixtures were put into the twin screw extruder (BP-8177, Dongguan Baopin International Precision Instrument Co., Ltd., Guangzhou, China) to get the granulation at 180 °C. And the granulation was put into the 3D printing consumables extruder to prepare filamentous materials (Figure 1) with a diameter (1.75 ± 0.05 mm), the printing temperature, layer height, nozzle diameter and filling rate are 180 °C, 0.1 mm, 0.5 mm and 100%.

### 2.3. Measurements and Characterization

Fourier transform infrared spectroscopy (FTIR) (Nicolet5700, Thermo Fisher Nicolet, Waltham, MA, USA) and X-Ray diffraction (XRD) were used to characterize the 3D printing materials. The FT-IR spectra were determined with KBr method. XRD patterns of the samples were obtained by a Polycrystalline X-ray diffractometer (Bruker AXS D8 Advance, Karlsruhe, Germany) and CuKα radiation (40 kV and 50 mA) was employed with 2θ varying between 5° and 40° at 5°/min. All the samples were heated to a maximum temperature of 180 °C and then cooled to 80 °C, both at 5 °C/min.

Melt flow rate (MFR) and rheology behavior were used to analyze the fluidity the 3D printing materials. MFR was tested by a melt flow indexer (XNR-400B, Chengde Dahua Testing Machine Co., Ltd., Chengde, China) and was used for different 3D printing samples at 180 °C and load 2.16 kg. The maximum torsional and equilibrium torque were measured to characterize rheology behavior of the samples with a torque rheometer (POLYLAB QC, Thermo Fisher Scientific, Shanghai, China) at 180 °C and 40 r/min.

The filamentary samples were printed into 80 × 10 × 4 mm^3^ blocks (GB/T 1843-2008, China) (Figure 1) by a 3D printer (WPC-500, Guanyu three dimensional Electronic Technology Co., Ltd., Qingdao, China) for the impact strength of each sample which were measured on a pendulum electronic impact testing machine (JB-300B, Jinan Heng Think Grand Instrument Co., Ltd., Jinan, China). All tests of each composition were repeated at least five times, and the average values were adopted. The microstructure of the impact fractured surfaces of each sample was performed in a field emission scanning electron microscope (FEI Sirion 200, Hongkong, China) operating at 3 kV. The fractured surfaces of impact section were sputtered with gold to avoid electrical charging during examination prior to processing.

## 3. Results and Discussion

### 3.1. Fourier Transform Infrared Spectroscopy

**Figure 2:**
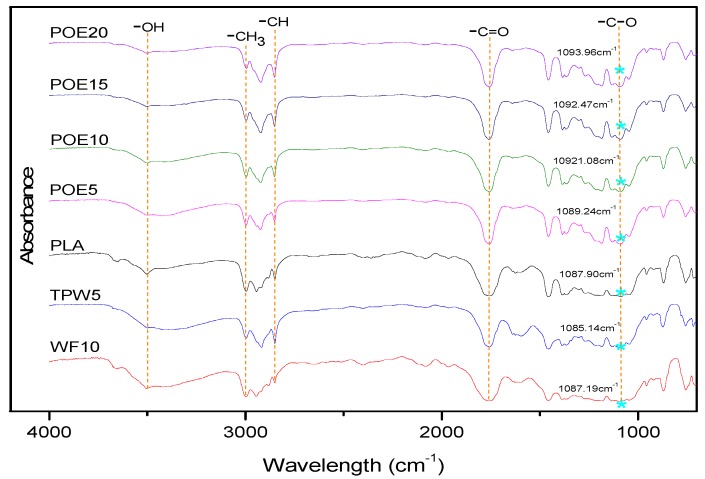
Fourier transform infrared spectra (FTIR) of 3D printing materials.

The FTIR spectra of the 3D printing material samples are presented in Figure 2. In the neat PLA spectrum, an asymmetrical –OH vibration is responsible for the peak at 3500 cm^−1^ [20]. Peaks at 3000~2900 cm^−1^ are due to asymmetrical CH_3_ telescopic vibrational absorption [21]. The peaks at 2900~2800 cm^−1^ can be attributed to asymmetric of CH stretching, and the distinctive symmetric peak at 1700 cm^−1^ is attributed to C=O carbonyl stretching [22]. Spectra for the other six samples were similar to the neat PLA, which means that almost all of the functional groups are contributed by PLA. But the relative intensity of these features varied, which was attributed to the variety in the weight fraction of PLA with corresponding variety in the amount of additives, in the composite samples. Moreover, it is interesting to note that the position of C–O stretching vibration peak has changed in different samples, which was often due to increased crystallinity during processing as result of thermal degradation. In addition, the shifts of the peaks also showed that wood flour and the lubricant have adverse effects on the toughness of the samples. But the POE could toughen the 3D printing materials because of the hydrogen bond interaction between the C=O of POE and the –OH of PLA. The POE could insert into and separate the PLA molecular chain, and reduce the molecular force, toughen the 3D printing materials by the hydrogen bond interaction [23].

### 3.2. X-ray Diffraction

**Figure 3:**
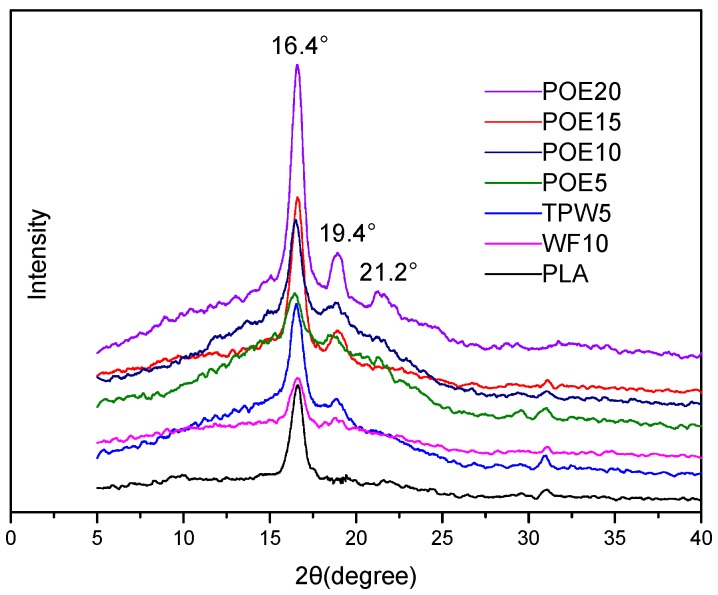
X-ray diffraction (XRD) spectra of 3D printing materials.

Figure 3 indicates the XRD spectra of all the 3D printing material samples. Except for a narrow peak at 2θ = 16.4°, PLA exhibits an amorphous nature and it can be considered as in a semi-crystalline phase [24]. The diffraction peak intensity of the composite increased with the addition of the poplar powder, which indicated that the poplar powder/PLA composite had an increase in crystallinity compared with PLA. The interaction between the poplar powder and the PLA matrix restricted the movement of the molecular chains, leading to the formation of a rigid interface and decreasing the interface compatibility of the composite [11]. And the presence of the poplar powder could act as the nucleation agent, initiates the growth of the crystalline phase of the material by nucleation. And the TPW5 curve is similar to WF10 curve, but the diffraction peak intensity of the composite increased with the addition of lubricant which may be because that the lubricant TPW604 resulted in the secondary crystallization. Besides, the addition of POE produced 2θ = 19.4° and 21.2° new crystal diffraction peaks. Although the positions of these peaks had not changed, the intensity of the diffraction peaks increased with the increase of POE which may be because that POE reduced the interaction between the molecular chains of PLA and improved the activity of PLA in the crystallization process. Therefore, it was beneficial to the orderly arrangement of PLA molecules [25].

### 3.3. Melt Flow Rate

**Figure 4:**
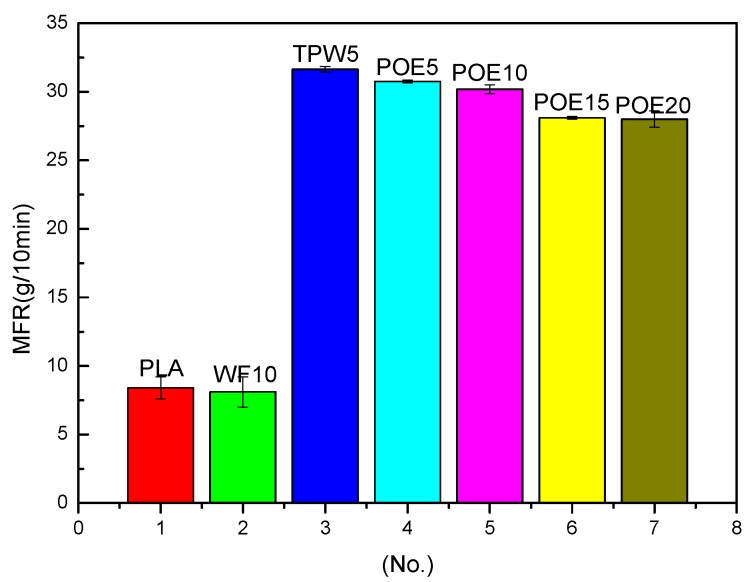
Melt flow rate (MFR) of 3D printing materials with different additives.

Figure 4 indicates the effects of wood flour, lubricant and flexibilizer on the MFR of 3D printing materials. As is shown in Figure 4, the effect of different additive on MFR of 3D printing materials is different. Obviously, the poplar powder decreased MFR of PLA, which is consistent with Rajabian [26] who thinks that fibers would reduce the MFR of polymers, and the more fibers content, the lower the MFR of polymer. Wood flour is not conducive to 3D printing. What is most noticeable is that TPW604 greatly improved the MFR of PLA, which is because TPW604 was compatible with PLA and increased the fluidity of PLA. And the lubricant would facilitate the 3D printing process. In addition, the trend from No. 3 to No. 7 shows a slow decline it is because the increase of POE content leads to the decrease of PLA matrix content and leads the poor fluidity of composites. But Figure 4 also showed that POE has little effect on MFR of the 3D printing material on the whole.

### 3.4. Torque Rheometry Analysis

**Figure 5:**
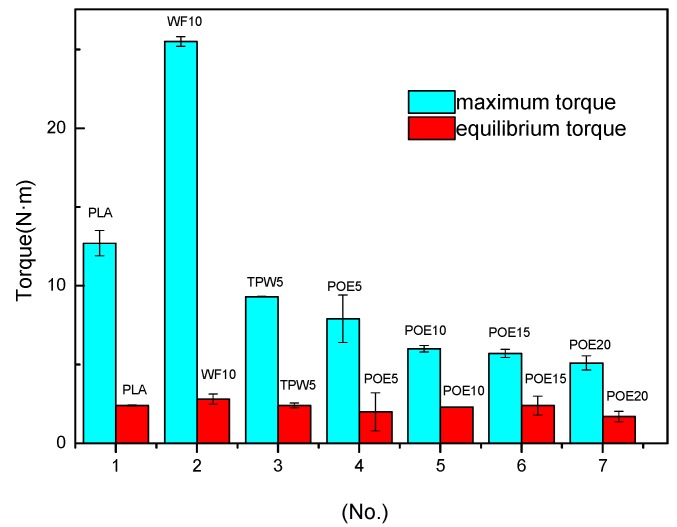
Maximum and equilibrium torque of different samples for 3D printing materials.

The maximum and equilibrium torque pattern of different samples for 3D printing materials are presented in Figure 5. Overall, there is no obvious wave in the equilibrium torque of different samples means that no matter wood flour, lubricant or toughening agent have little effect on the equilibrium torque of 3D printing materials. It can be observed from the pattern that poplar powder increased the maximum torque of PLA significantly because of the steric hindrance fillers introduced into the matrix and the poor compatibility between strongly polar wood fiber and non-polar PLA. As a rigid particle, wood flour has poor mobility, which seriously hindered the movement of chains in PLA, increased the viscosity of the melt, and decreased the fluidity of PLA. However, the lubricant TPW604 could increase the maximum torque of 3D printing materials effectively it is because the TPW604 reduced the friction between PLA and wood flour, which contributed to the flow of the melt. We can also see that the addition of POE can increase the maximum torque of 3D printing materials, and the higher the content, the lower the maximum torque. Toughening agent POE can reduce viscosity, improve fluidity and improve the processing performance of 3D printing materials. On the other hand, POE could prevent the agglomeration of wood flour, which was useful for the dispersion of wood flour in PLA evenly.

### 3.5. Impact Strength

**Figure 6:**
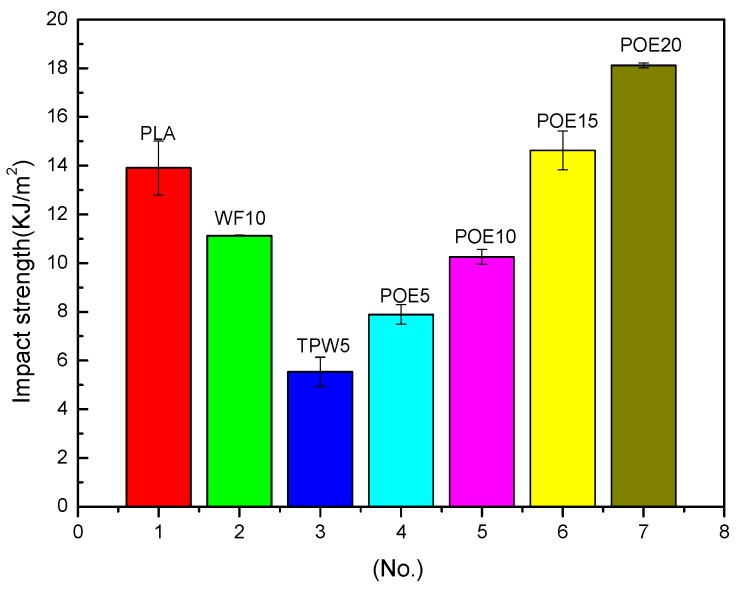
Impact strength of 3D printing materials.

The impact strength patterns of 3D printing materials are presented in Figure 6. The impact strength of a composite is dependent on factors such as particle dispersion, wetting, and infiltration of polymer in the particles. It can be observed from the pattern, the amount of poplar powder added in this experiment to neat PLA significantly decreased its impact strength, which is consistent to the effect of fiber on the impact strength of other polymers [27]. When the poplar powder is unevenly distributed in PLA, agglomeration phenomenon would occur easily, which could produce tiny voids in the composites, and lead to the stress concentration. As a result, the impact strength of the composites would decrease sharply. A proper amount of lubricant can improve the fluidity of melt, but excess lubricant could weaken the intermolecular force of the polymer such 5 wt %, and the ideal amount of lubricant is generally between 0.25% and 2% [28]. This could explain the negative effect of the lubricant TPW 604 on the impact strength of 3D printing materials. What is expected is that the toughening agent POE can effectively improve the toughness of 3D printing materials and the higher the content of POE, the higher the toughness. That is because the proper amount of POE is uniformly dispersed in PLA, which can form multiphase structure material with good interface with wood/PLA composites [29]. When the blends are subjected to external forces, POE as stress concentration points could lead to a large number of crazing and shear bands. With the branching of the craze around it, the stress at the end of the silver grain is reduced, which hinders the further expansion of the craze absorbs a large amount of impact energy, and greatly improve the toughness of the 3D printing materials [30]. At high temperature, POE could flow into and fill the void caused by wood flour in composites and weaken the stress concentration as fluid state. This may also explain the effect of POE on the impact strength of 3D printing materials.

### 3.6. Microstructure

**Figure 7:**
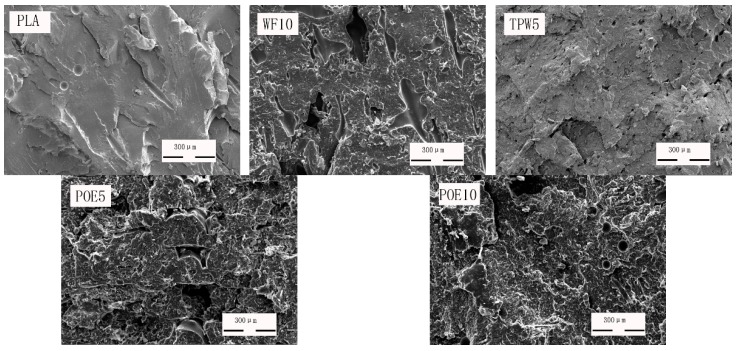

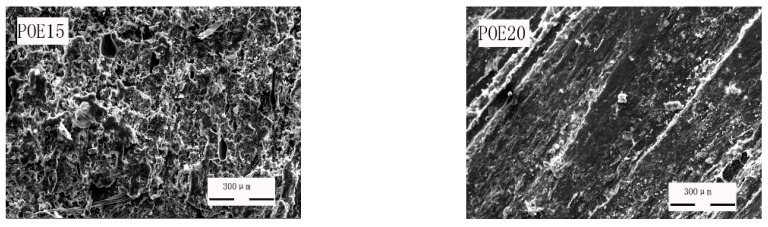
SEM images of the broken impact sections.

Scanning electron microscope (SEM) images of the broken impact sections of 3D printing materials are illustrated in Figure 7. The surface of pure PLA is smooth and uniform and there are almost no pores and voids in the matrix, indicating that there is a close bond between the PLA matrix. But the addition of poplar powder would have a negative impact on the interface of PLA. The section of the composites became rough and highly uneven, a large number of cracks appeared and the structure of PLA was destroyed because of the agglomeration of wood flour in the PLA matrix [31]. At high temperatures, lubricant TPW604 flowed into and filled the gaps of poplar powder/PLA composites as a fluid state, but still could weaken impact strength because of the brittleness of lubricant, which is why the surface is as smooth as PLA. What’s different is that the addition of POE has an important impact to the interface of 3D printing materials. When the raw materials were melted and mixed, the POE could form an interface layer between the wood flour and PLA, which can effectively transfer stress. So the interface adhesion was improved and the impact strength of 3D printing materials was enhanced [32].

## 4. Conclusions

In this study, new 3D printing materials were prepared with PLA and poplar powder. 3D printing materials were manufactured using 3D printing consumables extruder at 180 °C, lubricant and toughening agent were used to improve the fluidity and toughness of the 3D printing materials. Following are the main conclusions which can be drawn from the research: the addition of poplar powder was bad for the fluidity and toughness of PLA because of the agglomeration. Although the lubricant improved the fluidity, it reduced the impact strength of 3D printing materials. In addition, the effect of the toughening agent was up to the expectations. The POE not only could improve the fluidity and toughness of 3D printing materials, but also the higher the content, the better the property in a certain range because of the effect of POE on wood flour. As consideration, 3D printing materials prepared in this study not only could be applicable to 3D printing, but also be environmentally, friendly and promising in the field of printing.

## Figures and Tables

**Table 1:** The content of different ingredients in the formula (wt %).

No.	Polylactic Acid (PLA)	Poplar Powder	TPW604	Polyolefin Elastomer (POE)
1(PLA)	100	0	0	0
2(WF10)	90	10	0	0
3(TPW5)	85	10	5	0
4(POE5)	80	10	5	5
5(POE10)	75	10	5	10
6(POE15)	70	10	5	15
7(POE20)	65	10	5	20
